# The impact of feeding supplemental minerals to sheep on the return of micronutrients to pasture via urine and faeces

**DOI:** 10.1038/s41598-023-29717-3

**Published:** 2023-02-16

**Authors:** P.-T. Kao, H. Fleming, H. Warren, T. Darch, S. P. McGrath, H. L. Buss, M. R. F. Lee

**Affiliations:** 1 Rothamsted Research, North Wyke, Okehampton, EX20 2SB Devon UK; 2 Alltech Bioscience Centre, Sarney, Summerhill Road, Dunboyne, Co. Meath Ireland; 3 Rothamsted Research, Harpenden, AL5 2JQ Hertfordshire UK; 4 School of Earth Sciences, University of Bristol, Bristol, BS8 1RJ UK; 5 Harper Adams University, Newport, TF10 8NB Shropshire UK

**Keywords:** Element cycles, Biomechanics, Zoology, Environmental sciences

## Abstract

The form (organic versus inorganic) of minerals (Se, Zn, Cu and Mn), supplemented to sheep (Charolais × Suffolk-Mule (mean weight = 57 ± 2.9 kg) at two European industrial doses, on the return of micronutrients to pasture via nutrient partitioning and composition in sheep urine and faeces was investigated. This gave four treatments in total with 6 animals per treatment (n = 24). The form of the supplemented minerals did not influence the excretory partitioning of micronutrients (Se, Zn, Cu and Mn) between urine and faeces, nor on their concentrations in the excreta. The two doses trialed however, may influence the Se flux in the environment through altering the ratios of Se:P and Se:S ratios in the faeces and Se:S ratio in the urine. Administration of the mineral supplements also improved the retention of P in sheep reducing its excretion via urine. Although the concentrations of readily bioavailable micronutrients in the faeces were not affected by the mineral forms, there were differences in the more recalcitrant fractions of Se, Zn and Cu (as inferred via a sequential extraction) in faeces when different forms of supplemental minerals were offered. The potential impact of these differences on micronutrient flux in pasture requires further investigation.

## Introduction

Within grazing pasture systems, ruminant excreta are the major source of micronutrient inputs^1^. The composition of nutrients in urine and faeces can substantially impact the cycling of micronutrients in pasture after they are applied to soils. The concentrations of micronutrients in urine and faeces are related to their concentrations in the feed and status of the elemental micronutrients (minerals) required for health of the animal^2^. Grassland farmers in the UK are reported to use, on average, between two and three different methods to correct nutrient deficiencies in their livestock, which include licks, boluses, injections, supplementing water, supplementing feed, using (soil/foliar) fertilisers, and drenching^3^. However, because of the variable micronutrient concentrations in forages and the variable requirements at different growth stages of animal, the precise assessment of the degree of deficiency for an optimal supplementation is difficult^3^. Therefore, micronutrient supplements to livestock are often used prophylactically and routinely as part of standard practice, rather than strategically based on the nutrient level in feeds and/or the nutrition level of animals. Different chemical forms of minerals fed to animals can affect nutrient absorption efficiency in animals^4^. The greater absorption of the supplements is typically assumed to equate to reduced micronutrient loss from animal. However, the absorbed micronutrients can still be excreted through endogenous excretion, such as bile and sloughed epithelial cells^1^. It is not clear whether or not different forms (organic or inorganic) of the supplemental minerals have a significant impact on the excretion and partitioning of micronutrients between urine and faeces, and the subsequent bioavailability of micronutrients after the excreta are applied to soils.

It has been previously reported that Zn, Cu and Mn in sheep are mostly excreted via faeces, which includes undigested minerals, as well as endogenous excretions from metabolised minerals, such as pancreatic secretion of Zn and bile excretion of Cu and Mn^4–8^. However, studies reporting the impact of different forms of supplemental minerals on the partitioning of Zn, Cu and Mn between urine and faeces are rare. No significant difference in faecal excretion of Zn was found between ZnO, Zn-glycine, Zn-lysine, and Zn-methionine (at ca. 80 mg Zn day^−1^), yet higher urinary excretion of Zn was found in the treatments of ZnO and Zn-glycine over Zn-lysine and Zn-methionine^9^. There are limited studies investigating the effect of different chemical forms of supplemental Cu and Mn on their partitioning between excreta forms. A study investigating different forms of Mn and the excretion and absorption of Mn in lambs showed that there was no significant difference in the faecal excretion of Mn between the treatment of MnSO_4_ and Mn chelate of glycine hydrate^10^. For Se, a significant interaction effect was reported between diet (forage-based versus concentrate-based) and Se chemical form (Se-yeast versus Na_2_SeO_3_) on Se urine partitioning^11^. But, no significant influence of Se supplement forms (Na_2_SeO_3_, Se-yeast and Se-Met) on Se urine partitioning was also reported^12^. The feeding of supra-nutritional levels of Se has been reported to increase Se partitioning into urine^12–14^. Therefore, different doses of mineral supplementation adopted in different studies may explain contrasting results. In the current study, the two supplement dose levels used by the European feed industry were adopted to better reflect a likely level used on farms.

Nutrient balance (between macro and micro) in urine and faeces is also critical to micronutrient uptake by plants after urine and faeces are applied to soil^15^. The uptake of SeO_4_^2−^ and SeO_3_^2−^ by perennial ryegrass (*Lolium perenne* L.) are subject to competition with SO_4_^2−^ and PO_4_^3−^, respectively, due to their similar electron configuration of the outermost electron shells^16^. Whether or not the administration of different forms of supplemental minerals would have influence on the balance between Se and S and P requires further investigation.

The possible pathways through which the different form of supplemental minerals may impact micronutrient deposition to pasture via excreta and ultimately potential uptake by plants include altering: (1) micronutrient partitioning between urine and faeces; (2) concentrations or chemical form of micronutrients in urine and faeces; and (3) nutrient balance (micro and macro) in urine and faeces. To investigate these three potential impact pathways, 24 sheep were either given organic or inorganic forms of mineral supplements (Se, Zn, Cu, Mn) at two different doses adopted by the European feed industry for two weeks following a 2-week acclimatization period. The total amount and the concentrations of nutrients excreted in urine and faeces were subsequently determined. The chemical forms of Se, Zn, Cu and Mn in faeces were investigated via sequential extraction.

## Material and methods

### Treatments and experimental design

Concentrated pellets of animal feed (concentrates) containing either premixed inorganic minerals: selenite, zinc oxide, copper sulphate pentahydrate and manganese oxide, or organic minerals: selenised yeast (Selplex^®^, Alltech Inc., KY, USA) and Cu, Zn, Mn chelates of protein hydrolysate (Bioplex^®^, Alltech Inc., KY, USA) were given to castrated yearling male Charolais × Suffolk-Mule sheep. The premixed minerals were blended into the concentrate prior to pelleting, by a feed company (HJ Lea Oakes, UK), at two European feed industrial doses of inclusion. Table 1 shows the energy and nutrient contents of the concentrate feed reported by the feed company. There were in total four treatments: organic minerals at a higher (OH) or a lower (OL) inclusion and inorganic minerals at a higher (IH) or a lower (IL) inclusion. The doses of the OH and IH treatments were typically used by European industries based on the regulation of National Research Council of U.S.A.^17^. The doses of the OL and IL treatments were used by Alltech to be compliant to the administration of Selplex^®^ and Bioplex^®^. For Cu, Zn and Mn, the lower doses were 80% of the high doses. The lower dose for Se was 30% of the high dose treatment because the maximum permitted allowance of the inclusion of organic Se was 0.2 mg Se kg^−1^DM diet at 12% moisture under EU regulation^18^.

**Table 1:** Nutrient and energy content of the concentrate feed.

Components and nutrients		Control	OH	OL	IH	IL
Supplemental minerals (mg-element kg^−1^)	Selenium	–	0.6	0.2	0.6	0.2
	Copper	–	17	13	17	13
	Zinc	–	104	84	104	84
	Manganese	–	60	48	60	48
Metabolisable energy (MJ kg^−1^)		10.9	10.9	10.9	10.9	10.9
Crude ash (%)		7.59	7.60	7.63	7.58	7.58
Crude fibre (%)		7.77	7.76	7.76	7.76	7.76
Crude protein (%)		17.0	17.0	17.0	17.0	17.0
Dry matter (%)		88.0	88.0	88.0	88.0	88.0
Crude oils and fats (%)		3.65	3.65	3.65	3.65	3.65
Starch and sugar (%)		30.3	30.2	30.2	30.3	30.3
Starch (%)		22.9	22.9	22.9	22.9	22.9
Sugar (%)		7.35	7.35	7.35	7.35	7.35
Neutral detergent fibre (%)		20.8	20.7	20.7	20.7	20.7
Sodium (%)		0.45	0.45	0.47	0.47	0.47
Calcium (%)		1.11	1.12	1.11	1.12	1.12
Magnesium (%)		0.23	0.23	0.23	0.23	0.23
Salt (%)		1.30	1.30	1.35	1.35	1.35
Digestible undegradable protein (g kg^−1^)		42.4	42.4	42.4	42.4	42.6
Effective rumen degradable protein (g kg^−1^)		107	107	107	107	107
Vitamin A (IU kg^−1^)		–	8000	8000	8000	8000
Vitamin B12 (mcg kg^−1^)		–	60.0	60.0	60.0	60.0
Vitamin D3 (IU kg^−1^)		–	2000	2000	2000	2000
Vitamin E (IU kg^−1^)		–	10.0	10.0	10.0	10.0

*OH* organic minerals offered at higher doses, *OL* organic minerals offered at lower doses, *IH* inorganic minerals offered at higher doses, *IL* inorganic minerals offered at lower doses.

Sheep were offered the supplemented concentrates for two weeks along with big bale grass silage, sourced from a permanent pasture dominated by perennial ryegrass (*Lolium perenne*), at a 40:60 DM-based concentrate:silage ratio. Analytical methods and nutritional quality of the feed are reported in the supplementary material (Supplementary Figs. S1 and S2). The sheep (n = 24) were pre-weighed and condition scored (mean weight = 57 ± 2.9 kg; Body Condition Score (BCS) = 3.3 ± 0.20) and allocated into six blocks according to body weight to ensure that the mean value of body weight of the sheep was not significantly different across treatments. The sheep were individually penned and offered silage with one of the four different treatments of concentrate, and with drinking water (total hardness level = 15 mg calcium L^−1^) provided to individual troughs in the pens. The experiment was carried out at the Robert Orr Small Ruminant Facility at North Wyke, Rothamsted Research, using a Biocontrol System (BioControl^®^, As, Norway) for automatic feeding and data recording.

### Sheep acclimatization, feeding and monitoring of animal health

The sheep were brought into the facility two weeks before the start of supplementation for acclimatization. A control concentrate (without the additional premixed minerals) was offered as a wash-out and control basal period to all sheep for one week before the start of the supplementation period (day 0). During this pre-experiment period the control concentrate was slowly increased by ca. 100 g day^−1^ to achieve the 40:60 ratio of concentrate:silage (DM basis). The excreta collected on day 0 before the morning feeding was deemed the baseline of mineral excretion from the basal diet before the introduction of supplementation. The supplementary feeding started from day 0 when the control concentrate was replaced with the concentrate containing the targeted supplemental minerals (Table 1). The nutrient contents of the silage and the drinking water are provided in Supplementary Tables S1 and S2, respectively. All animals were weighed weekly prior to morning feeding and BCS assessed at the same time. The daily total intake (not including water) of Se, Zn, Cu and Mn from the basal diet and the mineral supplement is shown in Table 2.

**Table 2:** Total daily intake of Se, Cu, Zn and Mn from the silage, background concentrate and concentrate premix with percentage of total intake in parentheses.

Element	Treatment	Silage	Concentrate		Total intake
			Background	Premix of minerals	
Se (mg animal^−1^ day^−1^ ± SE)	IL	0.02 ± 0.001 (9%)	0.09 ± 0.005 (38%)	0.13 ± 0.007 (53%)	0.25
	IH	0.02 ± 0.001 (4%)	0.10 ± 0.008 (19%)	0.37 ± 0.029 (77%)	0.51
	OL	0.02 ± 0.002 (10%)	0.08 ± 0.007 (37%)	0.11 ± 0.010 (53%)	0.21
	OH	0.02 ± 0.001 (5%)	0.10 ± 0.005 (20%)	0.37 ± 0.020 (76%)	0.49
Cu (mg animal^−1^ day^−1^ ± SE)	IL	5.10 ± 0.349 (28%)	5.05 ± 0.268 (28%)	8.06 ± 0.427 (44%)	18.2
	IH	4.72 ± 0.388 (24%)	5.09 ± 0.403 (26%)	10.1 ± 0.80 (51%)	20.0
	OL	4.97 ± 0.436 (31%)	4.19 ± 0.381 (26%)	6.67 ± 0.607 (42%)	15.8
	OH	5.36 ± 0.276 (26%)	5.12 ± 0.283 (25%)	10.2 ± 0.57 (49%)	20.7
Zn (mg animal^−1^ day^−1^ ± SE)	IL	20.6 ± 1.45 (20%)	32.2 ± 1.70 (31%)	50.5 ± 2.68 (49%)	103
	IH	19.1 ± 1.57 (17%)	32.4 ± 2.57 (28%)	63.5 ± 5.04 (55%)	115
	OL	20.1 ± 1.76 (23%)	26.7 ± 2.43 (30%)	41.8 ± 3.81 (47%)	88.6
	OH	21.6 ± 1.11 (18%)	32.6 ± 1.80 (28%)	64.0 ± 3.53 (54%)	118
Mn (mg animal^−1^ day^−1^ ± SE)	IL	100 ± 7.1 (63%)	30.8 ± 1.63 (19%)	29.0 ± 1.54 (18%)	160
	IH	92.6 ± 7.61 (58%)	31.0 ± 2.46 (19%)	36.5 ± 2.90 (23%)	160
	OL	97.5 ± 8.57 (66%)	25.5 ± 2.32 (17%)	24.0 ± 2.19 (16%)	147
	OH	105 ± 5.4 (61%)	30.7 ± 1.73 (18%)	36.8 ± 2.03 (21%)	173

*OH* organic minerals offered at higher doses, *OL* organic minerals offered at lower doses, *IH* inorganic minerals offered at higher doses, *IL* inorganic minerals offered at lower doses.

### Sample collection and storage

An aliquot of 1–2 mL of drinking water was taken from the individual water troughs and bulked as one sample on a weekly basis; three replicates in total were taken at each sampling time. Approximately 100 g fresh weight silage was sampled from each feeding bin before the daily morning feed and bulked as one overall daily sample. Total daily urine and faecal samples were collected separately and bulked individually from each sheep before the morning feed. A 50 mL sample of urine and ca. 200 g of faeces was taken from the daily urine and faecal sample of each animal, respectively, for further chemical analysis. All the collected samples were stored at − 20 °C before sample preparation and analysis.

### Sample preparation and analyses

The drinking water samples were acidified in 5% (v/v) HNO_3_ before total nutrient analysis. An aliquot of 1 mL urine samples was filtered and diluted 20 times in 0.5% HNO_3_ and 1% methanol for total nutrient analysis. Faeces were oven-dried at 105 °C and at 80 °C for dry matter measurement and total nutrient analysis and sequential extraction, respectively. A preliminary test showed that there was no difference in the result total analysis of Se, Zn, Cu and Mn in faecal samples that were dried using methods between air drying, freeze-drying and drying at 80 °C (data not shown). Silage samples were freeze dried and ground for silage quality and total nutrient analysis. After drying, samples of faeces or silage for the total nutrient analysis were finely ground and 0.25 g sample was pre-digested in 3 mL HNO_3_ followed by a digestion in 3 mL ultra-pure water (18 MΩ) and 2 mL H_2_O_2_ at 175 °C for 10 min in a microwave digestion system (MARS, CEM Corporation^®^, USA).

Total nutrient analysis performed using ICP-OES (Perkin Elmer^®^ Optima 7300 V and Agilent^®^ 5900 SVDV) or ICP-MS (Perkin Elmer^®^ NexION 300X) depending on the concentrations of nutrients in the samples. A modified three-step sequential extraction procedure proposed by the Bureau Community of Reference (BCR)^19^ was used to extract Zn, Cu and Mn (Supplementary Table S3) from the faecal samples. For Se, another sequential extraction method^20^, originally used for extracting iodine (I) from soil, was revised and adopted here (Supplementary Table S4). The concentration of KH_2_PO_4_ used in step 2 was changed from 0.15 to 0.016 M for the extraction of ‘exchangeable Se’^21^. The analysis of silage quality included: pH, volatile fatty acids (VFAs), ammoniacal nitrogen and fibre. The latter included the quantification of modified acid detergent fibre (mADF), acid detergent fibre (ADF), neutral detergent fibre (NDF) and acid detergent lignin (ADL), of which the analytical methods are described in in the supplementary material and results in Supplementary Table S1. Nutritional quality data, other than micro-nutrients, of concentrate feed were provided by the feed company HJ Lea Oakes via proximate analysis (Table 1).

### Data calculation and statistical analysis

Total daily intake of Se, Zn, Cu and Mn (Table 2) was estimated from the total consumption and the mineral concentrations of the silage and the concentrate, not including water, on day 13. An ANOVA model (y ~ block + mineral form + supplementary dose + form × dose) was used to test the impact of the form and dose level and their interaction on the response variables, including nutrient concentrations, micronutrient chemical forms in faeces and nutrient partitioning in the urine and faeces. To test the influence of time on nutrient concentrations in urine and faeces, a modified ANOVA model (y ~ block + day) was applied. QQ-plots were performed, and outliers were removed to ensure that the residuals from the ANOVA models followed a normal distribution. The post-hoc test was performed by means of least significant difference (LSD) with a significant level of 95% (P < 0.05) referred by ‘significance’ in all contexts. All the statistical analyses were conducted in R^22^.

### Ethics approval

Animals were sourced from the institutes own farm and assessed daily for health and well-being, as determined by alertness, feed and water intake. All procedures (none of which required anaesthesia or euthanasia) were conducted in accordance with the United Kingdom Animal (Scientific Procedures) Act 1986, approved by institutional ethical review committees (Rothamsted Research, Animal Welfare and Ethical Review Board) and conducted under the authority of the Project Licence number P592D2677. The study is reported in accordance with ARRIVE guidelines (https://arriveguidelines.org).

## Results

### Nutrient partitioning in urine and faeces in sheep

The chemical form of the supplemental minerals did not have a significant impact on the excretion of the micronutrients and the macronutrients in urine or faeces, nor on their retention as percentage of total intake (Table 3). However, the supplementary dose level significantly influenced the excretion of Se in urine (P < 0.001) and faeces (P < 0.05) as percentage of Se intake and the retention of Se as percentage of intake (P < 0.001). An effect of supplementary dose level on Cu excretion in faeces (P < 0.05) and retention (P < 0.05) as percentage of total Cu intake was also observed. The supplementary dose level had no significant impact on the excretion and retention as percentage total intake of Zn, Mn, P and S. It should be noted that the calculated retention as percentage total intake of Zn in sheep was negative across all the treatments.

**Table 3:** Mineral excretion in urine and faeces and retention in sheep (Charolais × Suffolk-Mule) on day 14 as a % of the total element intake on day 13 (% w/w) and total balance results.

Elements	Variables	Treatments				P-values of the ANOVA test		
		IL	IH	OL	OH	Form	Dose	Form × dose
Se	Excretion to urine (% of total intake)	11.2 ± 1.39	9.42 ± 0.670	13.0 ± 0.33	7.80 ± 0.806	0.936	0.001***	0.064
	Excretion to faeces (% of total intake)	52.2 ± 2.27	45.5 ± 3.14	50.9 ± 2.34	43.3 ± 2.51	0.511	0.016*	0.872
	Retention (% of total intake)	36.6 ± 1.85	45.1 ± 3.11	36.1 ± 2.33	48.9 ± 2.14	0.478	< 0.001***	0.377
Zn	Excretion to urine (% of total intake)	6.62 ± 1.901	8.20 ± 1.819	8.89 ± 1.849	7.63 ± 1.102	0.525	0.905	0.298
	Excretion to faeces (% of total intake)	114 ± 10.9	111 ± 13.8	115 ± 9.4	96.9 ± 6.74	0.522	0.309	0.474
	Retention (% of total intake)	− 20.7 ± 12.55	− 19.1 ± 15.15	− 23.8 ± 9.54	− 4.54 ± 6.571	0.593	0.336	0.415
Cu	Excretion to urine (% of total intake)	0.39 ± 0.078	0.32 ± 0.038	0.43 ± 0.040	0.34 ± 0.037	0.493	0.055	0.906
	Excretion to faeces (% of total intake)	97.6 ± 4.33	89.0 ± 2.04	92.8 ± 2.06	79.4 ± 4.82	0.081	0.012*	0.538
	Retention (% of total intake)	1.99 ± 4.374	10.6 ± 2.02	6.74 ± 2.062	20.3 ± 4.84	0.083	0.012*	0.538
Mn	Excretion to urine (% of total intake)	0.11 ± 0.026	0.12 ± 0.017	0.12 ± 0.018	0.11 ± 0.019	0.891	0.981	0.762
	Excretion to faeces (% of total intake)	95.3 ± 2.55	91.1 ± 5.31	90.2 ± 5.74	85.9 ± 7.34	0.326	0.419	0.995
	Retention (% of total intake)	4.57 ± 2.562	8.76 ± 5.317	9.70 ± 5.744	14.0 ± 7.34	0.327	0.420	0.994
S	Excretion to urine (% of total intake)	55.8 ± 5.83	59.6 ± 3.52	59.5 ± 4.33	54.4 ± 3.46	0.818	0.860	0.232
	Excretion to faeces (% of total intake)	40.5 ± 1.19	42.5 ± 2.75	41.2 ± 2.43	38.7 ± 3.57	0.547	0.932	0.393
	Retention (% of total intake)	3.63 ± 5.358	− 2.17 ± 5.216	− 0.64 ± 5.546	6.89 ± 5.685	0.556	0.832	0.114
P	Excretion to urine (% of total intake)	0.19 ± 0.057	0.11 ± 0.028	0.07 ± 0.009	0.12 ± 0.027	0.189	0.734	0.112
	Excretion to faeces (% of total intake)	94.3 ± 4.31	94.6 ± 4.06	93.9 ± 3.53	83.5 ± 6.73	0.256	0.324	0.297
	Retention (% of total intake)	5.47 ± 4.310	5.25 ± 4.070	6.08 ± 3.523	16.4 ± 6.73	0.252	0.323	0.302

Significant values are in bold.

*OH* organic minerals offered at higher doses, *OL* organic minerals offered at lower doses, *IH* inorganic minerals offered at higher doses, *IL* inorganic minerals offered at lower doses. The symbol ‘*’ and ‘***’ indicate statistical significances of ANOVA test at p-value < 0.05 and < 0.001, respectively.

**Figure 1:**
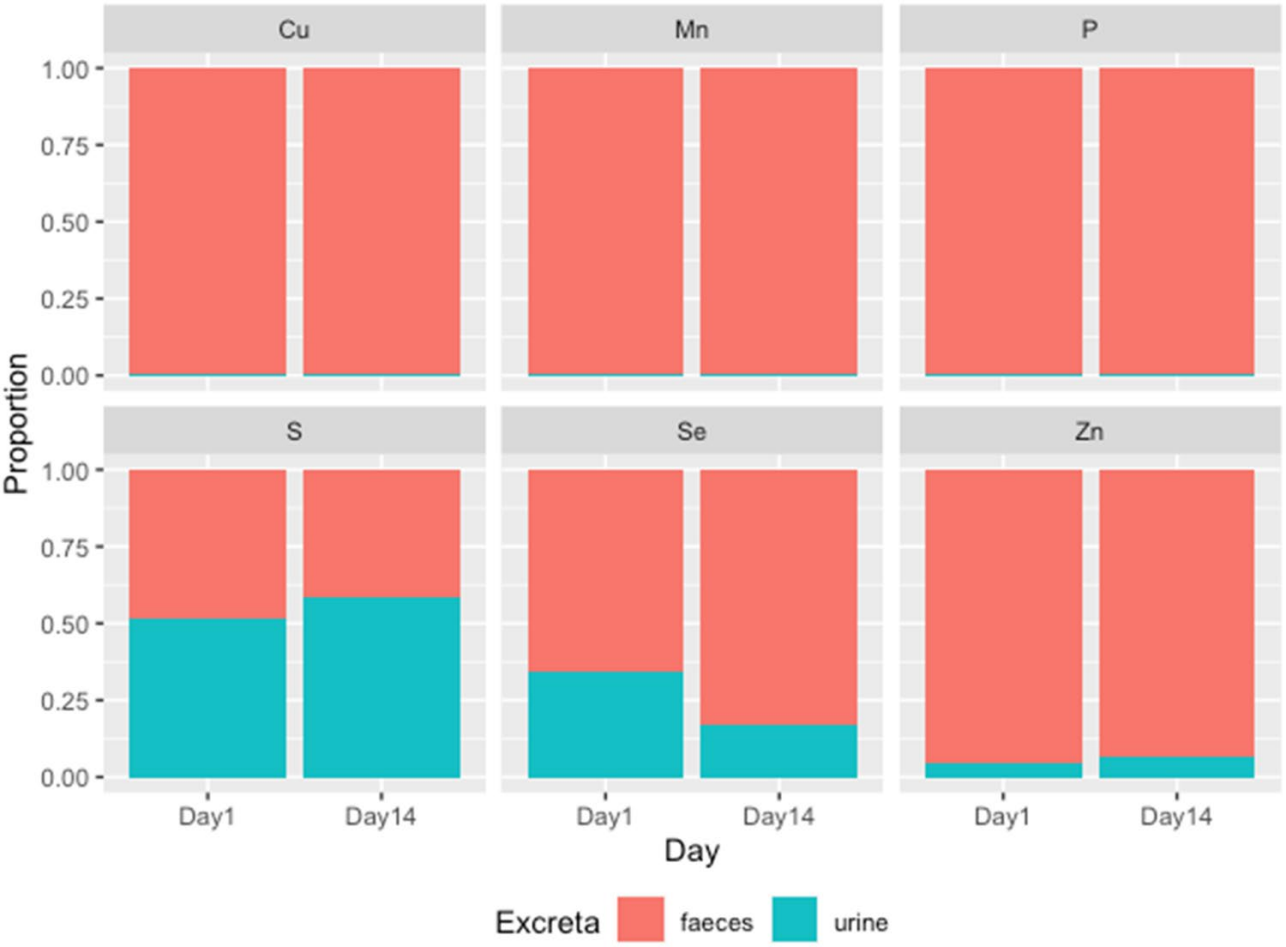
The proportion of mineral partitioning between urine and faeces of sheep (Charolais × Suffolk-Mule) on day 1 and day 14. Data are the average of the results across the four treatments.

Faeces was the dominant excretory route for Se, Zn, Cu, Mn and P, whereas urine was the major excretory route for S (Fig. 1). Over 90% of the Zn, Cu, Mn and P were excreted through faeces and over 60–80% of the Se was excreted through faeces. Unlike other nutrients, more than 50% of the S was excreted through urine. For Se there was an increase in the proportion excreted via faeces on day 14 (from 83%) compared with day 1 (66%). Conversely, there was a higher proportion of S excreted via urine on day 14 (58%) than on day 1(52%) (Fig. 1).

### The concentrations and predicted differences in chemical forms via sequential extraction of Se, Zn, Cu and Mn in faeces

**Figure 2:**
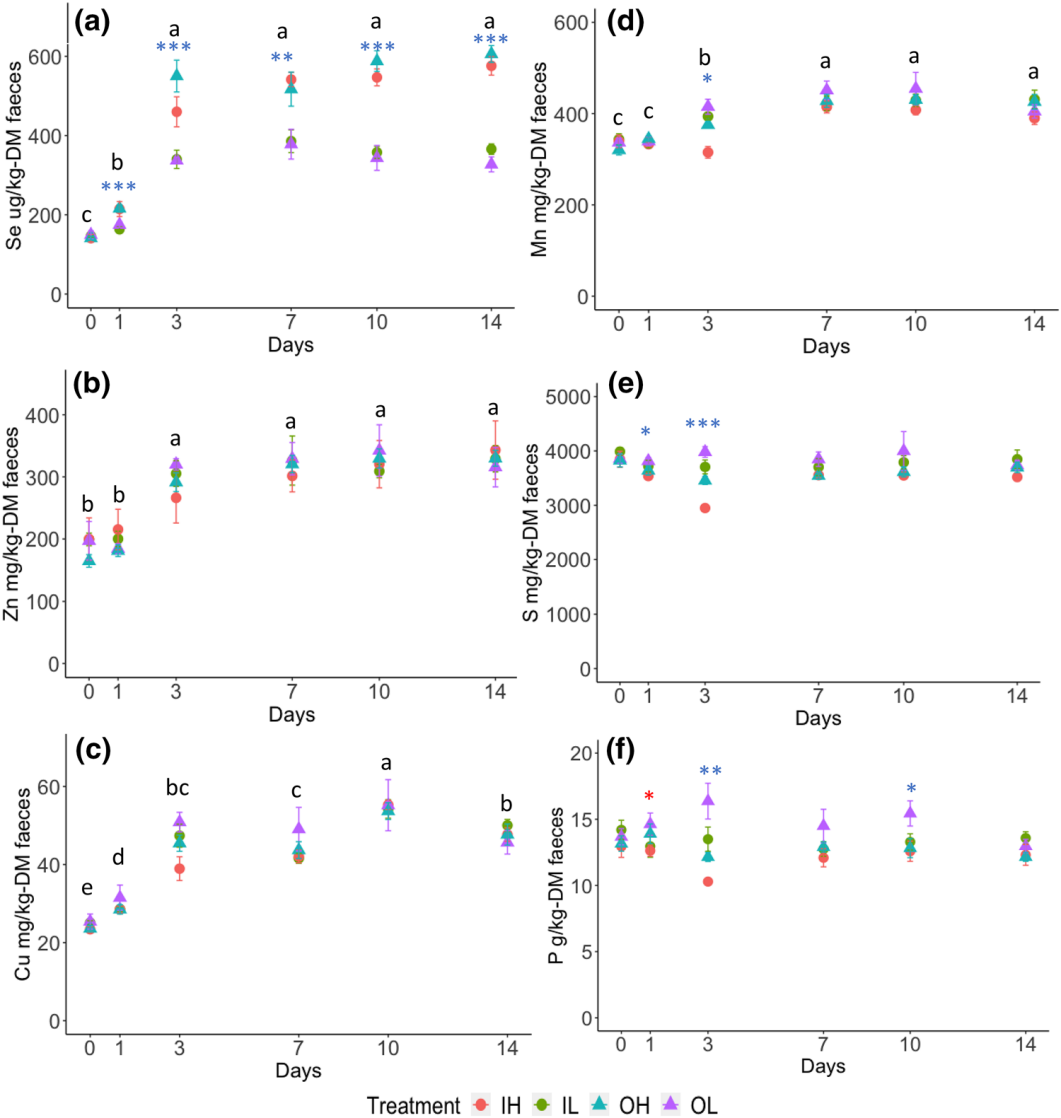
The concentrations of micronutrients and macronutrients in the faeces of sheep during the supplementary period. (**a**) Se (μg kg^−1^DM) in faeces (**b**) Zn (mg kg^−1^DM) in faeces (**c**) Cu (mg kg^−1^DM) in faeces (**d**) Mn (mg kg^−1^DM) in faeces (**e**) S (mg kg^−1^DM) in faeces (**f**) P (g kg^−1^DM) in faeces. The error bars are the standard errors (n = 6) of the samples. The colour of the symbol: ‘’, ‘’, ‘*’ represents the significant effect from supplement form (F1), supplementary dose (F2), the interaction of F1 and F2, respectively. The number of starts ‘*’, ‘**’ and ‘***’ indicate statistical significances of ANOVA test at p-value < 0.05, < 0.01, and < 0.001, respectively. The lowercase English letters represent the statistical results of post-hoc LSD test of temporal effect following a significant result of ANOVA test.

Micronutrient concentrations in the faeces of sheep fed organic supplements of Se, Zn, Cu and Mn followed a similar temporal trend to that of sheep fed the inorganic supplements. Se and Zn in faeces reached a plateau at day 3 after increasing concentrations (Fig. 2a,b). Although the concentration of Cu in faeces was variable across time, a trend towards stability after day 3 was observed (Fig. 2c). The Mn in faeces plateaued after day 7 after increasing concentrations (Fig. 2d). After the concentrations of nutrients in faeces had plateaued, the treatments with different chemical forms and dose levels of mineral supplements did not show a significant impact on the concentrations of nutrients in the faeces, other than for Se. The treatments of higher dose levels (OH and IH) resulted in significantly higher concentrations of Se in the faeces than the treatment of lower dose levels (OL and IL) (*P* < 0.001, Fig. 2a).

The results of sequential extractions showed that giving different forms of supplemental minerals to sheep had no significant impact on the chemical fractionation of Cu in the faeces (Table 4). For Zn, the inorganic mineral form of supplementation resulted in significantly higher Zn partitioning into the third fraction (oxidizable) (*P* = 0.0119, Table 4). However, the Zn in the third fraction accounted for less than 10% of the total Zn in the faecal sample. The supplementary dose and form had a significant impact on the chemical fractionation of Mn into the fourth fraction (residual) (*P* = 0.027 and 0.0415, respectively, Table 4). However, the Mn in the fourth fraction accounted for less than 1% of the total Mn. The supplementary form had a significant impact on the chemical fractionation of Se into the third fraction (bound to OM or sorbed to (Fe, Al)-hydroxides) (*P* < 0.001, Table 5). Giving an inorganic form of mineral supplement to the sheep resulted in more Se being extracted in the third step of sequential extraction than when an organic form of mineral supplement was offered. The treatments of the higher dose generally had higher concentrations of Se in all the four extracted fractions (*P* < 0.001, Table 5).

**Table 4:** Sequential extraction of Zn, Cu and Mn from faecal samples collected on day 14.

Extraction steps	Elements	IL	IH	OL	OH	P values		
		Mean ± SE (mg kg^−1^)				Form	Dose	Form × dose
Step 1: Exchangeable, water- and acid-soluble	Zn	115 ± 12.4	119 ± 20.0	113 ± 12.8	114 ± 7.9	0.767	0.852	0.900
	Cu	2.44 ± 0.093	2.67 ± 1.112	2.32 ± 0.172	2.43 ± 0.143	0.163	0.185	0.672
	Mn	307 ± 5.2	295 ± 8.0	310 ± 12.1	330 ± 16.0	0.102	0.732	0.165
Step 2: Reducible	Zn	138 ± 7.0	149 ± 23.2	147 ± 12.6	143 ± 4.0	0.902	0.745	0.539
	Cu	8.36 ± 0.500	7.91 ± 0.391	7.90 ± 0.456	7.76 ± 0.285	0.378	0.396	0.650
	Mn	64.8 ± 3.77	60.1 ± 2.96	62.1 ± 2.83	63.0 ± 2.27	0.988	0.529	0.355
Step 3: Oxidizable	Zn	15.8 ± 3.29	25.4 ± 2.32	20.0 ± 1.42	19.0 ± 1.01	0.012*	0.736	0.898
	Cu	22.1 ± 0.74	22.3 ± 0.85	21.1 ± 0.94	22.1 ± 0.89	0.565	0.551	0.701
	Mn	12.1 ± 2.16	9.99 ± 1.425	12.6 ± 0.88	10.1 ± 0.82	0.831	0.096	0.865
Step 4: Residual	Zn	5.81 ± 0.716	5.13 ± 0.374	4.57 ± 0.465	4.54 ± 0.261	0.054	0.428	0.463
	Cu	6.61 ± 0.143	6.90 ± 0.285	6.01 ± 0.893	6.70 ± 0.362	0.391	0.293	0.660
	Mn	2.46 ± 0.160	1.95 ± 0.149	1.98 ± 0.185	1.80 ± 0.130	0.042*	0.027*	0.263

Significant values are in bold.

*OH* organic minerals offered at higher doses, *OL* organic minerals offered at lower doses, *IH* inorganic minerals offered at higher doses, *IL* inorganic minerals offered at lower doses. The symbol ‘*’ indicate statistical significance of ANOVA test at p-value < 0.05.

**Table 5:** Sequential extraction of Se from faecal samples collected on day 14.

Extraction steps	IL	IH	OL	OH	P values		
	Mean ± SE (μg kg^−1^)				Form	Dose	Form × dose
Step 1: Water- and acid-soluble	54.7 ± 3.89	76.3 ± 5.65	51.2 ± 4.38	71.0 ± 5.00	0.470	< 0.001 ***	0.959
Step 2: Exchangeable	6.06 ± 0.730	11.2 ± 0.85	6.63 ± 0.431	10.8 ± 0.41	0.710	< 0.001 ***	0.317
Step 3: OM-bound and/or specific sorption on Fe/Al hydroxides	45.0 ± 2.57	74.4 ± 1.94	36.6 ± 2.50	67.3 ± 2.91	< 0.001 ***	< 0.001***	0.556
Step 4: Residual	56.2 ± 6.31	76.8 ± 8.76	61.3 ± 11.42	83.1 ± 8.56	0.589	0.038*	0.919

Significant values are in bold.

*OH* organic minerals offered at higher doses, *OL* organic minerals offered at lower doses, *IH* inorganic minerals offered at higher doses, *IL* inorganic minerals offered at lower doses. The symbol ‘*’ and ‘***’ indicate statistical significances of ANOVA test at p-value < 0.05 and < 0.001, respectively.

### The concentrations of Se, Zn, Cu and Mn in urine

**Figure 3:**
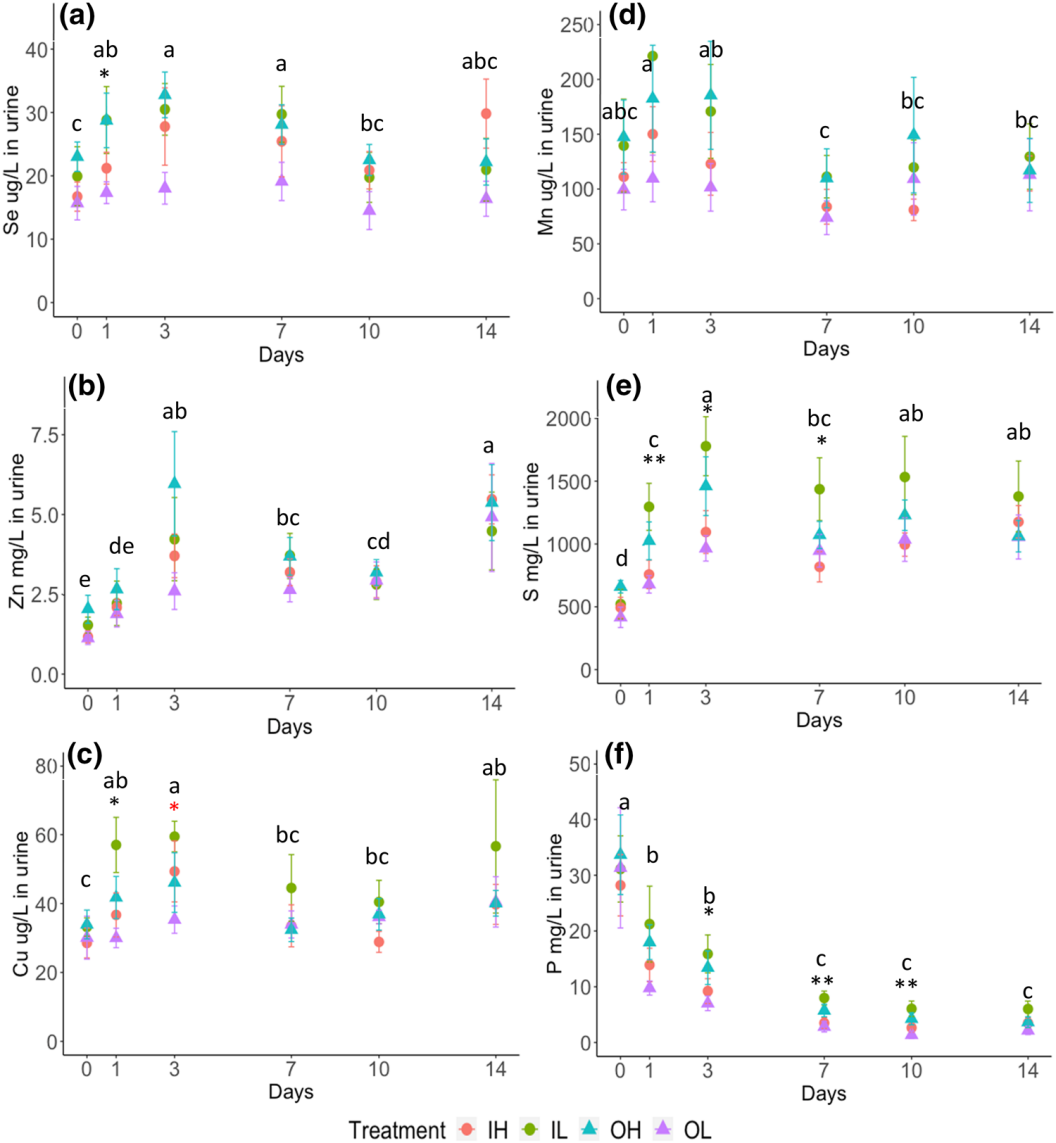
The concentrations of micronutrients and macronutrients in the urine of sheep during the supplementary period. (**a**) Se (μg L^−1^) in urine (**b**) Zn (mg L^−1^) in urine (**c**) Cu (μg L^−1^) in urine (**d**) Mn (μg L^−1^) in urine (**e**) S (mg L^−1^) in urine (**f**) P (mg L^−1^) in urine. The error bars are the standard errors (n = 6) of the samples. The colour of the symbol: ‘’, ‘’, ‘*’ represents the significant effect from supplement form (F1), supplementary dose (F2), the interaction of F1 and F2, respectively. The number of starts ‘*’ and ‘**’ indicate statistical significances of ANOVA test at p-value < 0.05 and < 0.01, respectively. The lowercase English letters represent the statistical results of post-hoc LSD test of temporal effect following a significant result of ANOVA test.

Although the temporal effect was significant on the concentrations of Se, Zn, Cu and Mn in urine, the concentrations did not appear to follow a clear trend through time and were variable through the supplementary period (Fig. 3a–d). The treatment effect was not significant for Zn and Mn throughout the experiment (Fig. 2b,d). For Se and Cu, although the treatment effect was significant on some days before day 3, there was no significant impact of the mineral supplement treatments or dose levels on the concentrations of Se and Cu after day 3 (Fig. 2a,c).

### The concentrations of S and P in the urine and faeces

The concentrations of S and P in the faeces of sheep did not appear to be affected by time of sampling (Fig. 2e,f). The concentrations of S and P in faeces remained stable across the supplementary period. The concentration of S in the urine reached a plateau after day 7 after increasing concentrations (Fig. 3e). Unlike other elements, the concentrations of P in urine steadily decreased from day 0 and reached the lowest concentrations on day 7 (Fig. 3f). The interaction between supplementary dose level and mineral form had a significant effect (*P* < 0.05) on the urine S and P on some days after the concentrations had plateaued (Fig. 3f). The mineral supplement form did not have a significant effect on the ratios of Se:P and Se:S in urine and faeces (Supplementary Table S5). The higher supplementary dose levels resulted in significantly (*P* < 0.001) higher ratios of Se:S and Se:P in faeces and Se:S in urine (Supplementary Table S5).

## Discussion

### The impact of chemical form of mineral supplement on micronutrient partitioning in urine and faeces

Urine and faeces, when applied to soils, are decomposed at different rates and release different amounts and forms of nutrients to the environment^23^. Therefore, in order to understand micronutrient cycling in pasture systems, investigations are critically needed into micronutrient partitioning between urine and faeces in grazing animals that are routinely given different forms and levels of supplemental minerals. In the present study, different chemical forms of the supplemental minerals (organic versus inorganic) offered at the different dose levels typically adopted by European feed industry had no impact on the partitioning and total excretion of Zn and Mn % of total intake in urine and faeces of sheep (Table 3). Although the different dose levels had a significant impact on the total excretion of Cu via faeces, the chemical form had no significant impact and faeces was still the dominant excretory route for Cu. This result is in agreement with previous studies that reported faeces as the major excretory route of Zn, Cu and Mn in sheep^4–8^.

It was found that the retention of Zn was negative across all the treatments. This most likely relates to the significant input of Zn from the drinking water (Supplementary Table S2), which was not included in the calculation of total intake. The concentration of Zn in the drinking water was significantly higher than the tap water and was provided in mg L^−1^ level, and was attributed to the galvanised pipes or troughs. The trough drinking water alone could be providing an estimated 2.14–11.9 mg Zn day^−1^, assuming a daily intake of 2–6 L water. The high provision of Zn intake from the basal diet (47–55%, Table 2) along with the Zn provision from the drinking water could have masked the effect of supplemented Zn on response variables. Similar masking effects could also have occurred for Mn, with ca. 80% provided from the basal diet (Table 2). This masking effect from the basal diet might be the reason why the dose level effect was only significant for Cu and Se, where the percentage from the basal diet was 50% and 30% at the higher dose treatments, respectively.

For Se, the study by Paiva, et al.^12^ indicated that the supplementary dose can be a determining factor as to whether the impact of supplemental mineral form is significant. At low Se supplementary doses (0.20–0.80 mg Se kg^−1^ DM or 0.23–1.04 mg Se day^−1^), Paiva et al.^12^ reported no significant effect of chemical form of Se supplements on the excretion of Se in faeces or urine. Whereas, at higher Se supplementary doses (1.4 mg-Se kg^−1^ DM or 1.68–1.98 mg Se day^−1^), there was a significant difference in faecal excretion of Se (inorganic Se > organic Se), but this was not mirrored in urine. However, in the current study, there was no significant interaction between dose level and form. The different chemical forms of the supplements given at the two different dose levels also had no significant impact on the partitioning of Se in urine and faeces (Table 3). The Se supplementary doses adopted in the current study were below 0.4 mg Se day^−1^ (Table 2), which is in the range of the ‘low Se supplementary levels’ in Paiva et al.^12^. Although supplementing Se at high levels (> 1.68 mg Se day^−1^) enabled the effect of different supplemented forms to be elucidated in the Paiva et al. study^12^, the European Commission restricts levels of supplementary organic Se to 0.2 mg Se kg^−1^ DM of complete intake at 12% moisture. In the current study, which wished to replicate current farming practice, this equated to ca. 0.25 mg Se d^−1^ depending on intake, at which the effect of the different chemical forms was not observed.

### The impact of chemical form of supplemental minerals on micronutrient concentrations and potential differences in chemical forms in faeces assessed by sequential extraction

Even though the different forms of supplemental minerals made no significant impact on the concentrations of Se, Zn, Cu and Mn in urine and faeces (Figs. 2, 3), this is not to say that the mineral form may not influence the chemical forms of the excreted Se, Zn, Cu and Mn in urine and faeces. Since faeces is the dominant excretory route of Se, Zn, Cu and Mn, the chemical forms of the elements in the faeces were investigated using sequential extractions. Each extracted fraction is operationally defined in the sense that each represents the amount of an element that can be extracted by a certain chemical solution. Although sequential extractions do not directly determine chemical speciation, they can provide an indication of the impact of treatments on the chemistry and bioavailability of the micronutrients excreted in the faeces. Elements that are extracted early in the process are generally weakly bound to the solid phase, and hence have greater potential mobility those released later^24^. Assuming that the bioavailability of extracted elements is a function of solubility and mobility, elements that are extracted by first two steps of the used sequential extraction are thought to be readily available to plants, and steps 3 and 4 progressively less so.

The results of the sequential extraction of faeces showed that supplemental mineral form had no significant impact on the extracted Se, Zn, Cu and Mn in the first two steps (Tables 4, 5). However, there were significant differences in the third step for Se and Zn extractions, and the fourth step for Mn extraction. These results imply that different forms (organic versus inorganic) of supplemental minerals would not impact the readily available Se, Zn, Cu and Mn after the faeces is applied to soil but may have a long-term effect on the ultimate flux of Se, Zn and Mn in the environment through influencing the less available fractions. The third and the fourth fractions of the sequential extractions are associated with micronutrients bound to organic matter (OM) and/or Fe/Al hydroxides, which are less mobile than the previous fractions. The release of the micronutrients from the OM and/or Fe/Al oxides after the faeces is applied to soil can be element-dependent based on the different affinity of Se, Zn, Cu and Mn species to OM and Fe/Al oxides, and the different geochemical reactions these elements may go through^1^. The bioavailability and the uptake of the releasing micronutrients can also be varied by different plants^25^. Further research is needed to understand the potential impact of supplemental mineral form on the long-term flux of Se, Zn and Mn in soil.

### Influence of the chemical form of supplemental minerals on the balance of Se, S and P in the urine and faeces

Availability of Se to plants is not solely associated with the total available Se in the environment. Previous studies have shown that the uptake of Se from soil can be decreased by elements that share the same nutrient transport mechanisms in plants. For example, application of S fertilizer to soil has been shown to decrease Se uptake by ryegrass, alfalfa (*Medicago sativa*) and wheat (*Triticum aestivum*)^26–28^. Fan et al.^29^ showed that the Se concentration in wheat grain was always low when sulphate fertiliser was applied. These observations can be attributed to the elemental antagonism between Se and S^26,28^, because SO_4_^2−^ and SeO_4_^2−^ are presumed to share the same transporter in plants^30^. In a solution culture trial with perennial ryegrass, a > 90% decrease in the uptake of SeO_4_^2−^ was observed in response to a tenfold increase in SO_4_^2−^, and a 30–50% decrease in the uptake of SeO_3_^2−^ in response to a tenfold increase in PO_4_^3−^ to the solution^16^. To reflect the potential antagonism between Se, S and P, their balance in urine and faeces were studied by calculating the ratios of Se:S and Se:P in the excreta (Supplementary Table S5).

No significant impact of supplemental mineral form was observed on either the ratios of Se:S and Se:P in excreta or on the readily available Se for plant uptake as shown in the sequential extraction (Table 5). Furthermore, the chemical forms and total concentrations of the P and S given to the sheep as well as the partitioning of P and S between urine and faeces were not significantly different across the treatments. However, the higher supplementary dose levels of Se resulted in higher ratios of Se:S and Se:P in the urine and faeces (Supplementary Table S5). The two doses adopted in the current study are doses that are typically used by the European feed industry. This result indicates that different doses of mineral supplements can significantly influence the ratios of Se:S and/or Se:P in sheep excreta and may, in turn, influence the Se flux in the environment. Studies regarding the potential influence of different Se:S and Se:P ratios in animal excreta on the flux of Se in pasture systems are therefore needed.

### A possible explanation for decreased P in urine from sheep offered mineral supplementation

The concentrations of P in urine of different treatments steadily decreased (Fig. 2f), but the concentrations of P in faeces remained consistent through time (Fig. 2f). The decrease with time in urinary P did not appear to be attributable to a variable P intake from silage, as the concentrations of P in silage provided during the supplementation period (day 1 to day 14) were not significantly different (Supplementary Table S3). The P concentration in urine has been shown to be related to the concentration of saliva P, and P partitioning between saliva and urine is influenced by the type of diet offered^4^. As the roughage content of the diet increases, the partition of initially absorbed P between salivary secretion and urinary excretion shifts towards the salivary route^4,31^. Salivation rate has been reported as the major controlling factor in urinary P excretion because decreasing salivation rate increases P concentration in plasma and results in more P being excreted via urine^32^. However, in the current study, a change in physical characteristics of the diet is unlikely to explain the decreasing P in urine as there was no significant difference in the ratio of silage:concentrate (Supplementary Figs. S1 and S2) nor silage digestibility (Supplementary Table S3) through time.

Despite being a small proportion of total body weight, micronutrients play critical roles in metabolic reactions^1^. For example, Zn plays a critical role in forming DNA-binding proteins that influence transcription, and hence cell replication^4^. The increased Zn intake can increase metabolism, which involves the use of P as part of adenosine triphosphate (ATP), and might in turn increase the use of P in sheep, leading to decreasing urinary P concentrations. Moreover, P is directly involved in Se metabolism, forming SePO_3_^3−^, an intermediate product in the synthesis of selenocysteine (Sec)^33,34^, which is also an ATP-requiring metabolic activity in animals. Therefore, the decreasing levels of P in the urine may be related to the mineral supplement intake, through improved mineral status of the animal which improves metabolism and hence P retention.

## Conclusion

This study investigated the potential influence of the chemical forms (organic versus inorganic) of the supplemented Se, Zn Cu and Mn on the nutrient partitioning, nutrient composition and nutrient balance in sheep urine and faeces, which may further influence the cycling of micronutrients in the environment. To reflect on-farm practices, two supplement doses of mineral supplementation used by the European industry were adopted. The form of the supplemented minerals at either industrial dose did not significantly impact on the concentrations of Se, Zn, Cu and Mn excreted, nor on their excretory partitioning between urine and faeces. Although the concentrations of readily bioavailable micronutrients in the faeces were not affected by the mineral forms, there were differences in the more recalcitrant fractions (via sequential extraction) of Se, Zn and Cu in faeces when different forms of supplemental minerals were given to sheep. The impact of these differences on micronutrient flux in pasture and the wider environment requires further investigation. The different supplemental doses also altered the ratios of Se:P and Se:S in the faeces and Se:S in the urine, which may affect the Se flux in the environment. Further study is also needed to investigate this potential influence. Finally, decreasing concentrations of P in the urine were observed when the sheep were given mineral supplements. This observation was attributed to a potential improved metabolism in the sheep given the mineral supplements and, in return, an improved retention of P. Whether or not the observation of a decreased P concentration in urine is reproducible in other mineral supplementation trials, and to what extent this might influence the P cycling in the systems requires further investigation.

## Supplementary Information

Supplementary Information.

## Data Availability

The dataset generated and analysed during the current study are available from the Rothamsted repository, https://doi.org/10.23637/rothamsted.98883.
