# Supplementary material for: The impact of feeding supplemental minerals to sheep on the return of micronutrients to pasture via urine and faeces
Source: Sci Rep. 2023 Feb 16;13:2747. doi: 10.1038/s41598-023-29717-3 (PMC9935513; doi:10.1038/s41598-023-29717-3)
Supplement: Supplementary file 1 — Supplementary Information. [file 41598_2023_29717_MOESM1_ESM.docx]

**Supplementary material**

**Paper Title:** The impact of feeding supplemental minerals to sheep on the return of micronutrients to pasture via urine and faeces

**Authors:**

P.-T. Kao^a^, H. Fleming^a^, H. Warren^b^, T. Darch^a^, S. P. McGrath^c^, H.L. Buss^d^, M.R.F. Lee^e^

^a^ Rothamsted Research, North Wyke, Okehampton, Devon, EX20 2SB, UK

^b^ Alltech Bioscience Centre, Sarney, Summerhill Road, Dunboyne, Co. Meath, Ireland

^c^ Rothamsted Research, Harpenden, Hertfordshire, AL5 2JQ, UK

^d^ University of Bristol, School of Earth Sciences, Bristol, BS8 1RJ, UK

^e^ Harper Adams University, Newport, Shropshire, TF10 8NB, UK

**Corresponding author**: Pei-Tzu Kao. email: b01601029@gmail.com

**Journal name**: Scientific Report

## Silage quality analysis

### Sample preparation and analysis of fibre component

The fibre component analysis included the quantification of modified acid detergent fibre (mADF), acid detergent fibre (ADF), neutral detergent fibre (NDF) and acid detergent lignin (ADL). Samples for the analysis of fibre composition were freeze-dried and coarsely ground with a grinder (Retech-SM-300). To prepare the samples for the analysis, two different subsamples of 0.45-0.50 g were weighed into two different filter bags. For NDF analysis, the filter bag ANKOM® F58 was used, while the bag ANKOM® F57 was used for the sequential analysis of mADF, ADF and ADL. The bags with the samples were sealed using a heat sealer within 4 mm of its open end. After this, they were placed in a beaker and soaked with enough acetone to cover the bags for 10 min. Afterwards, the acetone was poured out and the bags were placed on a wire rack to air-dry. The bags were then placed on bag suspender trays with up to three bags per tray.

To start the analysis of NDF, mADF, ADF and ADL, the bag suspender trays were stacked and placed in an ANKOM^2000^® Fibre Analyser. The analysis of the NDF fraction was conducted using a heat-stable bacterial alpha-amylase and sodium sulphate (Na_2_SO_4_), as specified in the methodology^1^. For the determination of mADF and ADF fractions, an acid detergent solution (ADS) was used for the extraction of the fractions. The solution was made following the methodology^2^ for ADF analysis. For the mADF analysis (first step in the sequence), the ADS was diluted with 1 N sulphuric acid (H_2_SO_4_) (1:1 v/v ratio). After the ADF analysis, bags were submerged in 72% H_2_SO_4_ for 3 h, as described in the method^3^ recommended by ANKOM Technology.

After each procedure described above, the samples were submerged again in acetone for 3-5 min and placed on a wire screen to air-dry before being oven-dried at 102 ± 2°C for 2-4 h. After this time, samples were placed into a desiccant pouch, prior to weighing, to cool down and to remain protected from moisture gain. The determination of each fraction was conducted by gravimetric analysis (weight before and after extraction), as described in the different methodologies for the different fibre fractions, and the values were expressed as % dry OM.

### Sample preparation and analysis of silage pH, volatile fatty acidss and ammoniacal nitrogen

For the analysis of silage pH, volatile fatty acids (VFA) and ammoniacal nitrogen, samples were kept at -20°C before analysis. For the analysis of silage pH, an aliquot of 10 g of the frozen silage and 90 mL of ultra-pure water (18 MΩ) were mixed in stomacher bag and processed through an ultra- stomacher (400 Circulator-Seward®) at 230 rpm for 2 min (according to the instrument setting). Afterwards, the pH value was measured from the supernatant of the stomached sample using a pH/ORP meter (Seven2Go, Mettler Toledo®) coupled with a pH microelectrode (InLad Micro, Mettler Toledo®).

For the analysis of VFAs and ammoniacal nitrogen, an aliquot of 20 g of the frozen silage and 100 mL of ultra-pure water (18 MΩ) were mixed in a stomacher bag. After gently agitating the bag to ensure the water mixed well with the silage, the bag was then sealed and stored at 4°C overnight. On the next day, the extract was filtered through Whatman No.1 filter paper in a cold room (4°C). The filtered extract was stored at -20°C before the analysis. Analysis of VFAs was carried out by HPLC (1260 infinity-Agilent®) with an Agilent Hi-PLEX H^+^ column at 40°C and the eluent was 0.005 M H_2_SO_4_ at a flow rate = 0.6 mL/min. The VFAs were detected using a diode array detector (DAD) at wavelength = 210 nm. The peaks were identified by retention times and quantified against a range of the individual standards. The ammoniacal nitrogen was analysed using a photometric analyzer (Aquakem 250, Thermo Scientific®).

**Table S1.** Analyses of silage bales used during the experiment

| Item  (mean ± SE; n=3) | | Different bales of silage for different feeding dates | | | | | *P*-value |
| --- | --- | --- | --- | --- | --- | --- | --- |
|  |  | 12 to 11 days  before day 0 | 10 to 6 days  before day 0 | 5 days  before day 0 | Day 1 to day 7 | Day 8 to day 14 |  |
| pH | | 4.63 ± 0.006^b^ | 4.40 ± 0.009^d^ | 4.56 ± 0.009^c^ | 4.69 ± 0.018^a^ | 4.54 ± 0.006^c^ | **<0.001***** |
| Volatile fatty acids  (g kg-DM^-1^) | Lactic acid | 48.5 ± 3.16 | 69.0 ± 0.75 | 41.8 ± 9.90 | 47.0 ± 3.78 | 64.7 ± 1.20 | 0.227 |
|  | Acetic acid | 13.2 ± 0.75 | 19.0 ± 0.55 | 13.9 ± 2.85 | 12.3 ± 1.27 | 19.4 ± 0.71 | 0.248 |
|  | Propionic Acid | N.D. | N.D. | 0.229 ± 0.2286 | 0.112 ± 0.0791 | N.D. | 0.568 |
|  | Isobutyric acid | N.D. | N.D. | N.D. | N.D. | N.D. | - |
|  | Butyric acid | 5.71 ± 0.170 | 0.487 ± 0.0266 | 0.053 ± 0.0383 | 10.3 ± 0.93 | 0.569 ± 0.0605 | 0.575 |
|  | Valeric acid | N.D. | N.D. | N.D. | N.D. | N.D. | - |
| Ammonium (mg L^-1^) | | 214 ± 2.4 | 211 ± 1.4 | 208 ± 13.8 | 184 ± 11.8 | 196 ± 2.4 | 0.122 |
| Fibre compositions  (% of dry OM) | Neutral detergent fibre | 51.5 ± 0.33 | 51.9 ± 0.36 | 51.0 ± 0.32 | 52.8 ± 0.53 | 51.3 ± 0.62 | **0.109** |
|  | Modified acid detergent fibre | 29.4 ± 0.18^c^ | 30.1 ± 0.24^a^ | 29.4 ± 0.08^bc^ | 30.0 ± 0.14^ab^ | 29.1 ± 0.23^c^ | **0.016*** |
|  | Acid detergent fibre | 27.9 ± 0.03^ab^ | 28.3 ± 0.14^a^ | 27.4 ± 0.01^b^ | 28.3 ± 0.16^a^ | 27.5 ± 0.30^b^ | **0.007**** |
|  | Acid detergent lignin | 3.33 ± 0.443 | 2.97 ± 0.135 | 3.03 ± 0.293 | 3.84 ± 0.514 | 3.23 ± 0.405 | 0.544 |
| Cadmium (μg kg-DM^-1^) | | 3.99 ± 0.033^bc^ | 4.84 ± 0.331^a^ | 4.45 ± 0.040^ab^ | 3.61 ± 0.027^c^ | 4.60 ± 0.081^a^ | **0.002**** |
| Copper (mg kg-DM^-1^) | | 6.37 ± 0.068^bc^ | 6.82 ± 0.080^ab^ | 6.22 ± 0.365^c^ | 7.29 ± 0.051^a^ | 6.71 ± 0.043^bc^ | **0.010*** |
| Iron (mg kg-DM^-1^) | | 425 ± 8.0^b^ | 498 ± 2.8^a^ | 260 ± 0.4^e^ | 318 ± 0.7^d^ | 355 ± 3.7^c^ | **<0.001***** |
| Manganese (mg kg-DM^-1^) | | 133 ± 2.2^a^ | 131 ± 0.8^a^ | 110 ± 0.5^c^ | 125 ± 1.1^b^ | 121 ± 0.6^b^ | **<0.001***** |
| Molybdenum (mg kg-DM^-1^) | | 0.84 ± 0.003^c^ | 0.61 ± 0.003^d^ | 0.93 ± 0.006^a^ | 0.83 ± 0.001^c^ | 0.88 ± 0.005^b^ | **<0.001***** |
| Phosphorous (g kg-DM^-1^) | | 3.01 ± 0.027^a^ | 2.79 ± 0.018^c^ | 2.94 ± 0.016^b^ | 3.02 ± 0.017^a^ | 3.04 ± 0.027^a^ | **<0.001***** |
| Sulphur (g kg-DM^-1^) | | 2.37 ± 0.007^b^ | 2.52 ± 0.028^a^ | 2.37 ± 0.012^b^ | 2.41 ± 0.016^b^ | 2.45 ± 0.047^ab^ | **0.013*** |
| Selenium (μg kg-DM^-1^) | | 10.2 ± 0.45^a^ | 8.43 ± 1.111^a^ | 8.42 ± 0.632^a^ | 5.13 ± 0.738^b^ | 9.69 ± 0.249^a^ | **0.008**** |
| Zinc (mg kg-DM^-1^) | | 24.0 ± 0.45^a^ | 23.7 ± 0.46^b^ | 20.7 ± 0.36^a^ | 23.3 ± 0.28^a^ | 24.3 ± 0.44^a^ | **<0.001***** |

(N.D.: not detectable in the analyte; the lowercase letters behind numbers represent the results of LSD test post a significant result in general ANOVA test).

**Table S2.** Mineral contents in the drinking water for sheep collected on different experimental days from the trough and mineral contents of the tap water in the sheep facility

| Element  (mean ± SE, n=3) | Day 0 | Day 7 | Day 14 | Tap water  (background) | (Pr>F) | Limit of detection (LOD) |
| --- | --- | --- | --- | --- | --- | --- |
| Se (µg L^-1^) | 0.061 ± 0.0162 | 0.039 ± 0.0087 | 0.019 ± 0.0058 | 0.020 ± 0.0097 | 0.074 | 0.020 |
| Mn (µg L^-1^) | 2.37 ± 0.216^a^ | 1.89 ± 0.034^b^ | 2.54 ± 0.010^a^ | 0.23 ± 0.015^c^ | **<0.001***** | 0.050 |
| Cu (µg L^-1^) | 3.42 ± 0.187^b^ | 4.29 ± 0.565^b^ | 3.78 ± 0.388^b^ | 6.08 ± 0.255^a^ | **0.005**** | 0.050 |
| Zn (mg L^-1^) | 1.07 ± 0.047^c^ | 1.18 ± 0.036^b^ | 1.98 ± 0.025^a^ | 0.021 ± 0.002^d^ | **<0.001***** | 0.010 |
| Ca (mg L^-1^) | 7.27 ± 0.892^b^ | 8.56 ± 0.066^ab^ | 8.25 ± 0.034^b^ | 9.85 ± 0.104^a^ | **0.023*** | 0.020 |
| Cd (µg L^-1^) | 0.122 ± 0.0438^a^ | 0.030 ± 0.0101^b^ | 0.018 ± 0.0047^b^ | < LOD^b^ | **0.018*** | 0.010 |
| Fe (mg L^-1^) | 0.006 ± 0.0059^a^ | 0.012 ± 0.0032^a^ | 0.005 ± 0.0022^a^ | < LOD^b^ | **<0.001***** | 0.020 |
| Mo (µg L^-1^) | 0.041 ± 0.0121 | 0.033 ± 0.0039 | 0.049 ± 0.0094 | 0.046 ± 0.0027 | 0.548 | 0.010 |
| Na (mg L^-1^) | 7.85 ± 0.975 | 8.69 ± 0.053 | 8.90 ± 0.030 | 8.05 ± 0.015 | 0.417 | 0.020 |
| P (mg L^-1^) | 1.09 ± 0.024^a^ | 1.11 ± 0.007^a^ | 1.01 ± 0.012^b^ | 0.825 ± 0.0386^c^ | **<0.001***** | 0.020 |
| S (mg L^-1^) | 6.11 ± 0.646 | 7.07 ± 0.035 | 7.05 ± 0.026 | 6.67 ± 0.050 | 0.207 | 0.020 |

Different lowercase letters after numbers in the same row represent a significant LSD at p-value<0.05 in general ANOVA test.

Table S3. The extraction reagents in the revised BCR sequential extraction procedure

| Steps | Reagents | Nominal target phase(s) |
| --- | --- | --- |
| 1 | 0.11 M CH_3_COOH | Exchangeable, water- and acid-soluble |
| 2 | 0.5 M NH_2_OH-HCl at pH=1.5 | Reducible |
| 3 | H_2_O_2_ (85°C) then 1 M CH_3_COONH_4_ | Oxidizable |
| 4 | (revised) HNO_3_/H_2_O_2_ acid digestion | Residual |

Table S4. The extraction reagents used in the sequential extraction procedure for selenium

| Steps | Reagents | Nominal target phase(s) |
| --- | --- | --- |
| 1 | 0.01 M KNO_3_ | Water- and acid- soluble |
| 2 | (revised) 0.016 M KH_2_PO_4_ | Exchangeable |
| 3 | 10% TMAH | OM-bound a/o specific sorption on Fe/Al hydroxides |
| 4 | HNO_3_/H_2_O_2_ acid digestion | Residual |

**Table S5.** The concentration ratios of Se:P and Se:S in the urine and faeces of sheep (Charolais x Suffolk-Mule) on day 0 and day 14 and the result of ANOVA test across treatments

| Excreta | Ratios | Treatment | | | | *P*-values | | |
| --- | --- | --- | --- | --- | --- | --- | --- | --- |
|  |  | IL | IH | OL | OH | F1 | F2 | F1 x F2 |
| Urine | Day 0 Se:P | 1.21x10^-3^ | 6.51x10^-4^ | 8.30x10^-4^ | 8.27x10^-4^ | 0.172 | 0.769 | 0.423 |
|  | Day 14 Se:P | 6.17x10^-3^ | 1.08x10^-2^ | 9.40 x10^-3^ | 7.76 x10^-3^ | 0.964 | 0.533 | 0.199 |
|  | Day 0 Se:S | 3.83x10^-5^ | 3.58x10^-5^ | 4.05x10^-5^ | 3.55 x10^-5^ | 0.460 | 0.811 | 0.358 |
|  | Day 14 Se:S | 1.47x10^-5^ | 2.48x10^-5^ | 1.55x10^-5^ | 2.05x10^-5^ | 0.335 | **<0.001***** | 0.166 |
| Faeces | Day 0 Se:P | 1.05x10^-5^ | 1.09x10^-5^ | 1.09x10^-5^ | 1.08x10^-5^ | 0.909 | 0.770 | 0.722 |
|  | Day 14 Se:P | 2.69x10^-5^ | 4.73x10^-5^ | 2.52x10^-5^ | 4.98x10^-5^ | 0.701 | **<0.001***** | 0.060 |
|  | Day 0 Se:S | 3.68x10^-5^ | 3.67x10^-5^ | 3.89x10^-5^ | 3.71x10^-5^ | 0.473 | 0.532 | 0.613 |
|  | Day 14 Se:S | 9.55x10^-5^ | 1.63x10^-4^ | 8.74x10^-5^ | 1.64x10^-4^ | 0.282 | **<0.001***** | 0.241 |
| Faeces : Urine | Day 0 Se:P | 0.009 | 0.017 | 0.013 | 0.013 | - | - | - |
|  | Day 14 Se:P | 0.004 | 0.004 | 0.003 | 0.006 | - | - | - |
|  | Day 0 Se:S | 0.962 | 1.023 | 0.960 | 1.044 | - | - | - |
|  | Day 14 Se:S | 6.521 | 6.583 | 5.646 | 8.010 | - | - | - |


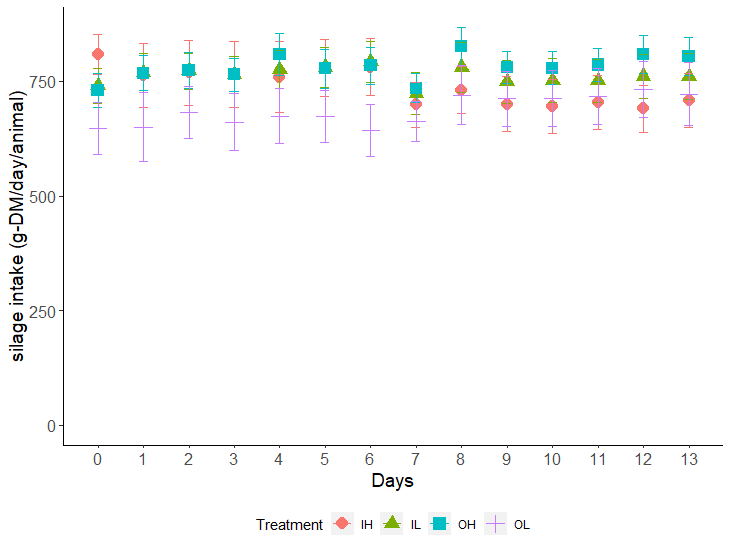

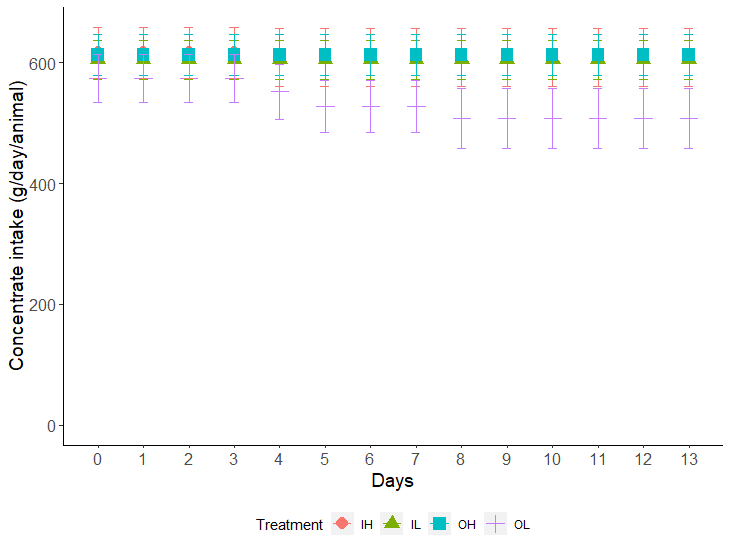


**Figure S1.** Daily silage intake (g-DM d^-1^animal^-1^) of sheep (Charolais x Suffolk-Mule) by treatments. The error bars are the calculated standard errors (n=6).

**Figure S2.** Daily concentrate intake (g d^-1^ animal^-1^) of sheep (Charolais x Suffolk-Mule) by treatments. The error bars are the calculated standard errors (n=6).

Reference

1 ANKOM Technology. Neutral Detergent Fiber in Feeds - Filter Bag Technique (for A2000 and A2000I) at <https://www.ankom.com/sites/default/files/document-files/Method_13_NDF_A2000.pdf>

2 ANKOM Technology. Acid Detergent Fiber in Feeds - Filter Bag Technique (for A2000 and A2000I) at <https://www.ankom.com/sites/default/files/document-files/Method_12_ADF_A2000.pdf>

3 ANKOM Technology. Method 8 – Determining Acid Detergent Lignin in beakers. at <https://www.ankom.com/sites/default/files/document-files/Method_8_Lignin_in_beakers_0.pdf>
